# Longitudinal Strain Is a Marker of Microvascular Obstruction and Infarct Size in Patients with Acute ST-Segment Elevation Myocardial Infarction

**DOI:** 10.1371/journal.pone.0086959

**Published:** 2014-01-28

**Authors:** Loïc Bière, Erwan Donal, Gwenola Terrien, Gaëlle Kervio, Serge Willoteaux, Alain Furber, Fabrice Prunier

**Affiliations:** 1 L’UNAM Université, Angers, France; 2 Université Angers, UPRES EA3860, Laboratoire Cardioprotection, Remodelage et Thrombose, CHU Angers, Service de Cardiologie, Angers, France; 3 Service de Cardiologie et CIC-IT 804, CHU de Rennes & Laboratoire Traitement du Signal et de l’Image, INSERM U1099, Rennes, France; 4 Université Angers, UPRES EA3860, Laboratoire Cardioprotection, Remodelage et Thrombose, CHU Angers, Service de Radiologie, Angers, France; Scuola Superiore Sant’Anna, Italy

## Abstract

**Objectives:**

We assessed the value of speckle tracking imaging performed early after a first ST-segment elevation myocardial infarction (STEMI) in order to predict infarct size and functional recovery at 3-month follow-up.

**Methods:**

44 patients with STEMI who underwent revascularization within 12 h of symptom onset were prospectively enrolled. Echocardiography was performed 3.9±1.2 days after myocardial reperfusion, assessing circumferential (CGS), radial (RGS), and longitudinal global (GLS) strains. Late gadolinium-enhanced cardiac magnetic imaging (CMR), for assessing cardiac function, infarct size, and microvascular obstruction (MVO), was conducted 5.6±2.5 days and 99.4±4.6 days after myocardial reperfusion.

**Results:**

GLS was evaluable in 97% of the patients, while CGS and RGS could be assessed in 85%. Infarct size significantly correlated with GLS (R = 0.601, p<0.001), RGS (R = −0.405, p = 0.010), CGS (R = 0.526, p = 0.001), ejection fraction (R = −0.699, p<0.001), wall motion score index (WMSI) (R = 0.539, p = 0.001), and left atrial volume (R = 0.510, p<0.001). Baseline ejection fraction and GLS were independent predictors of 3-month infarct size. MVO mass significantly correlated with GLS (R = 0.376, p = 0.010), WMSI (R = 0.387, p = 0.011), and ejection fraction (R = −0.389, p = 0.011). In multivariate analysis, GLS was the only independent predictor of MVO mass (p = 0.015). Longitudinal strain >−6.0% within the infarcted area exhibited 96% specificity and 61% sensitivity for predicting the persistence of akinesia (≥3 segments) at 3-month follow-up.

**Conclusions:**

Speckle-tracking strain imaging performed early after a STEMI is easy-to-use as a marker for persistent akinetic territories at 3 months. In addition, GLS correlated significantly with MVO and final infarct size, both parameters being relevant post-MI prognostic factors, usually obtained via CMR.

## Introduction

Echocardiography is a useful tool for risk stratification and prognosis assessment following acute myocardial infarction (AMI). Several echocardiographic parameters, such as left ventricular (LV) volume, ejection fraction (EF), wall motion score index (WMSI), presence of mitral regurgitation, and left atrial volume, have been shown to provide prognostic information [1]–[4]. LV volume and EF are the primary means for assessing myocardial systolic function and myocardial damage after AMI. Nevertheless, it must be taken into account that these indices are global and load-dependent. The development of cardiac magnetic resonance imaging (CMR) with the tagging approach and echocardiography with the speckle-tracking strain imaging has provided additional tools to assess global and regional functions according to myocardial fiber orientation and position within the myocardial thickness [5]–[7]. As a result, longitudinal, radial, and circumferential functions can be distinctively assessed. Using speckle-tracking imaging, several studies have demonstrated the usefulness of longitudinal and circumferential strains in differentiating between sub-endocardial and transmural AMI, and assessing post-AMI prognosis [7]–[9].

CMR is currently considered to be the most reliable method for determining microvascular obstruction (MVO) in the first days after reperfusion [10] and for measuring accurately infarct size a few weeks later [11], [12], both parameters being well-established prognosticators [11], [13], [14]. However, CMR accessibility is limited, whereas echocardiography is readily available.

In the present study, we sought to prospectively assess the value of speckle tracking imaging performed within the first days after successful reperfusion in ST-segment elevation myocardial infarction (STEMI) patients in order to predict initial microvascular obstruction (MVO) and infarct size at a later time point.

## Method

Patients with STEMI admitted to the Angers university hospital were prospectively enrolled. Inclusion criteria were as follows: primary or rescue percutaneous coronary intervention (PCI) for first STEMI within 12 hours of symptom onset; age above 18 years; culprit coronary artery with proximal occlusion, *i.e.,* proximal or mid-left anterior descending coronary artery, proximal dominant circumflex coronary artery, or proximal right coronary artery; thrombosis in myocardial infarction (TIMI)-flow Grade 0 or 1 prior to PCI, and successful revascularisation with TIMI-flow Grade 2 or 3 after stenting. Diagnosis of STEMI was defined by chest pain for at least 30 minutes, ST-segment elevation ≥0.1 mV in at least two or more limb leads, or ST-segment elevation ≥0.2 mV in two or more contiguous precordial leads. Exclusion criteria were cardiogenic shock, history of myocardial infarction or aorto-coronary bypass surgery, contraindication to CMR and cardiac arrest before PCI.

Baseline echocardiography was performed within 5 days after myocardial reperfusion. CMR was performed at baseline, within 10 days after myocardial reperfusion in order to assess MVO, with the examination repeated at 3-month follow-up in order to quantify infarct size and infarct transmurality.

The protocol was approved by the Institutional ethics committee at the University Hospital of Angers (France), and the study was conducted in accordance with the Declaration of Helsinki and French regulatory requirements. Prior to being included into the study, the patients gave their written informed consent.

### Echocardiography

Images were obtained in the left lateral decubitus position with a commercially available VIVID 7 system (GE Healthcare, Horten, Norway) using a 2.5 MHz transducer at a depth of 14 to 16 cm. Standard data on bi-dimensional echocardiography was collected according to American Society of Echocardiography (ASE) guidelines [15], with LV size evaluated by M-mode on a parasternal long axis view, and wall motion scored using a 16-segment LV model as follows: 1 = normokinetic, 2 = hypokinetic, 3 = akinetic, and 4 = dyskinetic. LV and left atrial volumes were estimated using the biplane Simpson’s method from apical four-chamber and two-chamber views. Aortic stenosis and mitral regurgitation were quantified [15].

**Figure 1:**
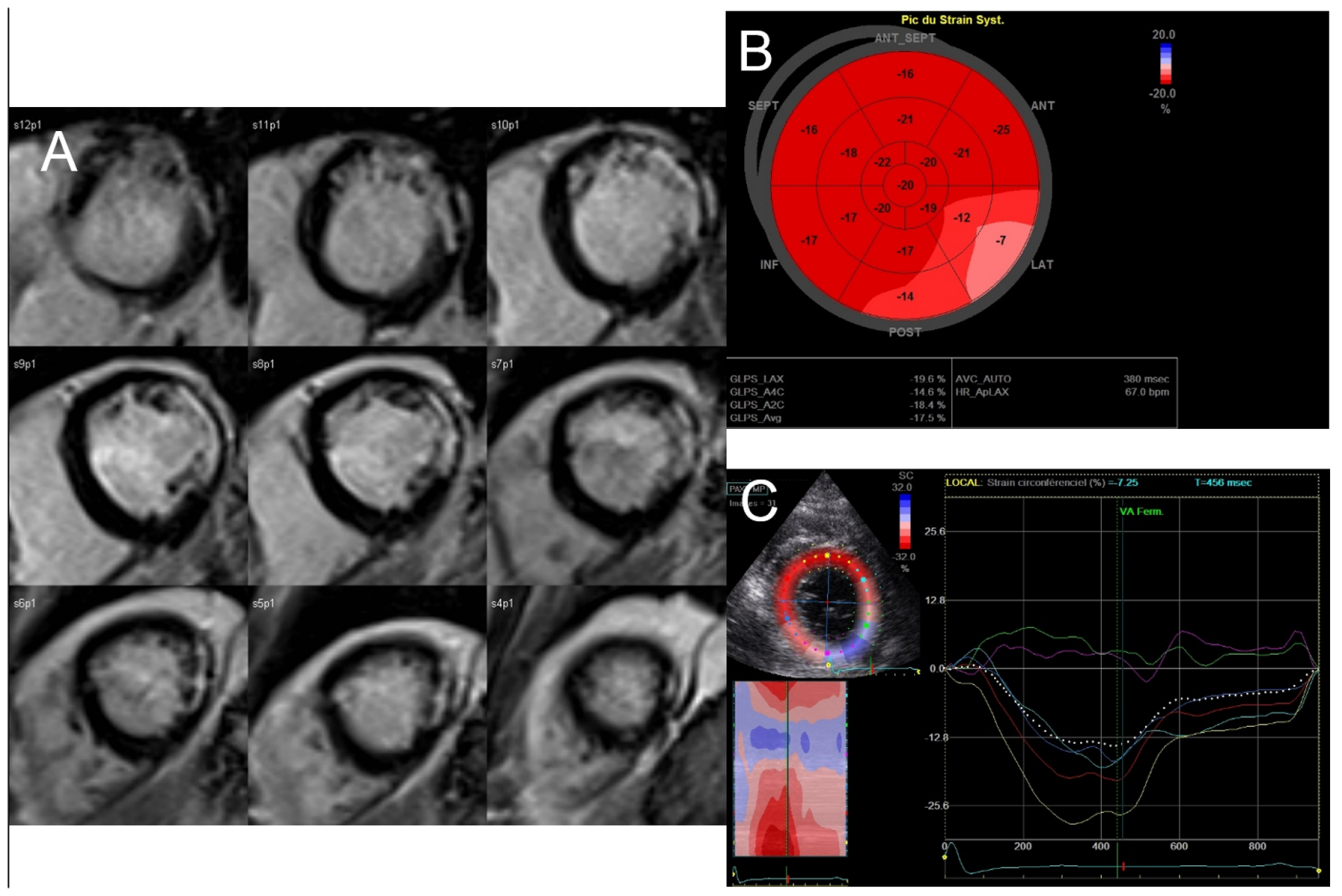
A case of lateral infarction related to the occlusion of the proximal circumflex coronary artery. A, CMR late gadolinium hyperenhancement slices of left ventricular short axis showing non transmural extension of myocardial infarct in the lateral zone; B, longitudinal global strain reprensented on bull’s eye view; it illustrates the correspondance between the late gadolimium enhancement (A) and the blunted longitudinal strain of the basal segments of the antero-lateral and infero-lateral left ventricular walls; C, the same blunted strain of the basal antero-lateral and infero-lateral left ventricular walls is observed in parasternal view when considering the circumferential function.

**Figure 2:**
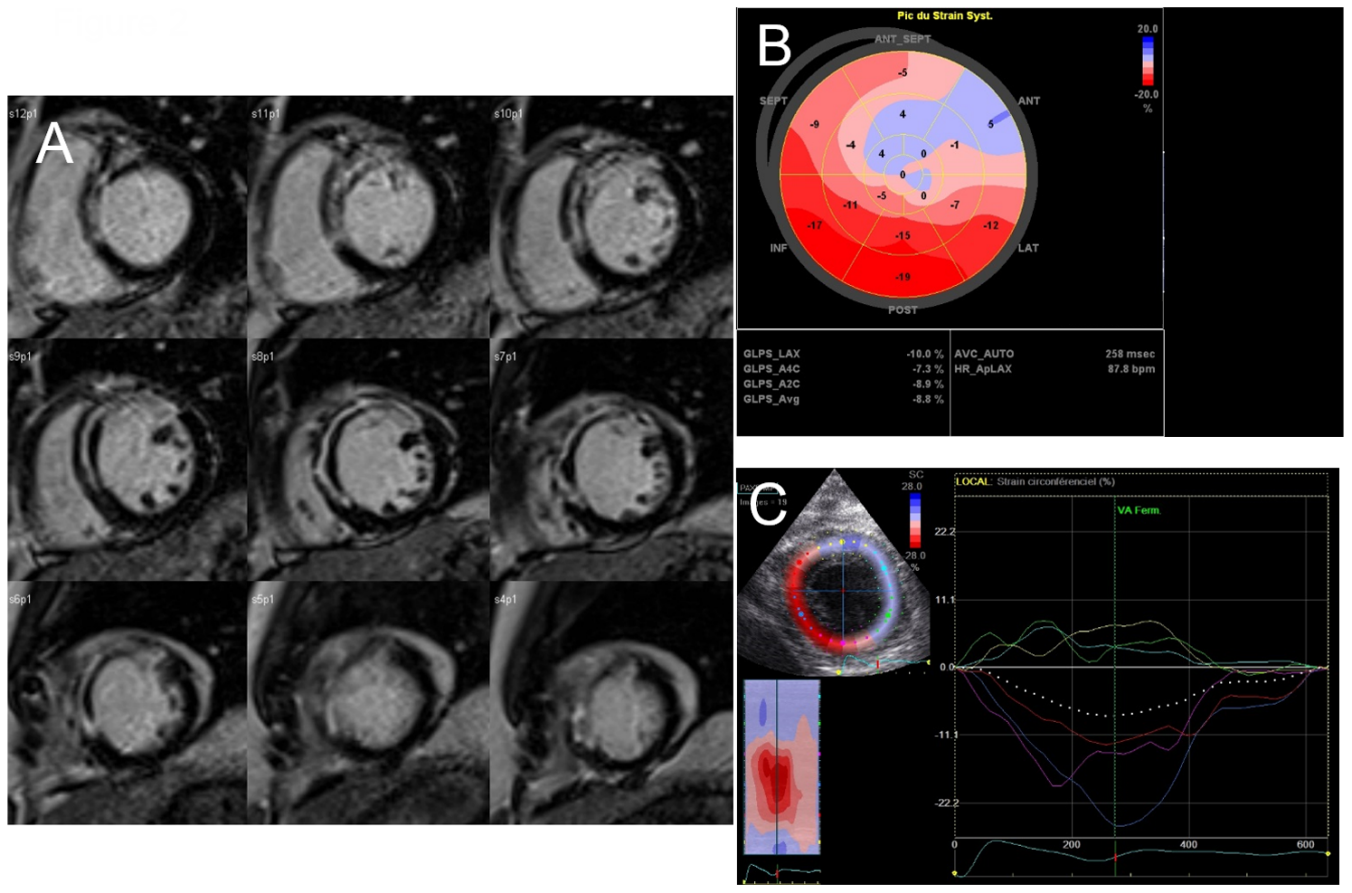
A case of anterior infarction related to the occlusion of the proximal left anterior descending coronary artery. A, CMR late gadolinium hyperenhancement slices of left ventricular short axis showing transmural extension of myocardial infarct in anterior and antero-septal zones and central hypoenhancement defining microvascular obstruction; B, longitudinal global strain represented on bull’s eye view; there is a severe dyskinesia (blue color) of the anterior wall; C, dyskinesia is also observed in parasternal view when considering the circumferential deformation of this severe transmural infarction.

For 2D-strain analysis, apical four-, three-, and two-chamber views, and parasternal short axis view at papillary muscle level were stored in cine-loop format, triggered to the QRS complex during one heart cycle with frame rates between 55 and 70 frames/sec. These cine loops were then analyzed off-line on the EchoPAC (GE healthcare, Horten, Norway) system, based on frame-by-frame tracking of natural acoustic markers [16]. Peak systolic longitudinal strain (LS) was determined for each of the 16 segments, and longitudinal global strain was then calculated. Peak radial and circumferential systolic strains were determined from papillary muscle level short axis view, with mean values computed. All acquisitions and analyses were carried out by a single experienced cardiologist blinded to the CMR results (Figures 1 & 2).

For 2D-strain analysis, inter-observer and intra-observer agreements were determined based on recordings obtained from twenty random patients.

### Cardiac Magnetic Resonance Imaging

CMR scans were performed with 1.5-T imager (Avanto, Siemens, Erlangen, Germany) using an 8-element phased-array cardiac receiver coil. Localization was carried out using breath-hold real-time and steady-state free precession images of true anatomical axes of the heart.

LV function was assessed by means of cine imaging using a segmented steady-state free precession pulse sequence in multiple short-axis, long-axis, and four-chamber views covering the entire LV. Typical in-plane resolution was 1.6×1.9 mm, with a 7.0 mm section thickness and the following parameters: repetition time (TR)/echo time (TE): 2.6 ms/1.3 ms; flip angle: 80°; matrix: 256×208; temporal resolution: 35–50 ms. Receiver bandwidth was set to 930 Hz/px and adapted to the shorter echo spacing (3.1 ms).

Late gadolinium enhancement (LGE) was performed 10–12 minutes following initiation of gadoterate meglumine (DOTAREM, Guerbet, Aulnay-sous-Bois, France), administered in cumulative dose calculated at 0.2 mmol/kg of body weight, along with a two-dimensional segmented inversion-recovery gradient-echo pulse sequence. Typical in-plane resolution was 1.68×1.68 mm, with a 7.0 mm section thickness and the following parameters: TE: 4.66 ms; flip angle: 30°; image acquisition triggered at every other heartbeat; matrix: 256×208. Inversion time was set to null the normal viable myocardium signal and typically ranged from 240 ms to 300 ms.

All deidentified images were analyzed at the central core laboratory by two experienced observers who were blinded to the patient data. Commercially available software (QmassMR 7.1, the Netherlands) was used for the analysis. Endocardial and epicardial borders were outlined manually on all end-diastolic and end-systolic short-axis cine slices. LV end-systolic and end-diastolic volumes, EF, and LV myocardial mass were then calculated in a standard manner.

The recommended 17-segment system was employed [17], with the two blinded observers’ consensus required.

LV segmental motion was assessed visually using a three-grade scale: 0 = normal; 1 = hypokinesia; 2 = akinesia, dyskinesia, or systolic expansion.

Transmural myocardial LGE extension was assessed visually. The segmental extent of LGE was assessed as follows: 0 = 0%; 1 = 1%–25%; 2 = 26%–50%; 3 = 51%–75%; 4 = 76%–100% hyperenhancement. We considered myocardium non viable if LGE exceeded 75% of mural extent. LGE area was manually defined on all segments, and infarct size was defined as the total LGE mass. Microvascular obstruction (MVO) was identified by the presence of a central hypoenhancement within the bright signal [10], and the MVO area was manually traced on all segments. In cases where no MVO was present, MVO mass was set at zero. For CMR parameter analysis, including LV volumes, EF, mass, LGE mass, and MVO mass, inter-observer agreements were determined based on data gathered from 20 random patients.

### Infarcted Area Analysis

A segment was considered as part of the infarcted area if LGE was present in more than 50% of the circumferential extent for that segment [18]. Mean LGE segmental extent in the infarcted area was calculated, with a value >75% required for the diagnosis of transmural infarction.

The mean value of longitudinal strain was calculated within the infarcted area (infarct LS) so as in the remote myocardium area.

### Statistical Analysis

Data was presented as mean±standard deviation or median (25^th^;75^th^ percentiles) in cases of non-normal distribution, with categorical data expressed as frequencies and percentages. We compared baseline with 3-month CMR data using McNemar test or a Chi-squared test, as appropriate. Baseline echocardiographic and CMR data obtained at 3-month follow-up was compared using Pearson’s correlation test. P-values <0.05 were considered statistically significant.

For multivariate analyses, echocardiographic data was tested by a descending step-by-step linear regression analysis, including variables with p values <0.05. The linear relation between dependent and independent variables was tested, as was the normal distribution of the residuals on PP plots. Analysis was performed using SPSS version 15.0 for Windows (SPPS, Inc, Chicago, IL).

We calculated the specificity, sensitivity, as well as positive and negative values of different parameters for predicting myocardial contractility recovery at 3-months (absence of recovery was defined by ≥3 akinetic segments at 3 month). ROC curves were generated.

Inter-observer agreement was studied in a blinded fashion using echocardiographic and CMR scans randomly selected from 20 patients, which were analyzed on EchoPac and QmassMR software by two different observers. Intra-observer agreement was also tested in a blinded fashion using 20 speckle tracking examinations, which were analyzed at two different times on EchoPac. Inter-and intra-observer agreements were assessed using the Bland and Altman method [19], and intraclass correlation coefficients (ICC) [20] were calculated. For radial and circumferential strain reproducibility analysis, two examinations were not evaluable due to lack of echogenicity on parasternal view.

## Results

In total, 44 patients were included in the study between February 2009 and March 2010. Of these, three were excluded from analysis, one because of surgically treated significant ischemic mitral regurgitation at 1-month follow-up, another because of significant aortic stenosis, and the remaining patient because of pace-maker implantation prior to the second CMR. Characteristics of the remaining 41 patients are summarized in Table 1. Of note, two patients with acute anterior STEMI presented small inferobasal, previously unknown infarctions. Echocardiographies were conducted 3.9±1.2 days and CMR 5.6±2.5 days and 99.4±4.6 days following myocardial reperfusion, with echocardiographic and CMR data summarized in Table 2.

**Table 1:** Characteristics of the patient population (n = 41).

Variable	
Age , y	57±12
Male, % (n)	82 (34)
Smoking, % (n)	56 (23)
Diabetes, % (n)	19 (8)
Hypertension, % (n)	39(16)
Family history of CAD, % (n)	41 (17)
Dyslipidemia, % (n)	56 (23)
Primary PCI, % (n)	80 (33)
Time onset of symptoms to PCI (min)	226 (176.5;303.7)
Culprit coronary artery: LAD/LCx/RCA (%)	66/17/17
Peak CPK, UI/L	1845 (1196;3149)
Medications at 3 months including aspirin, statins, clopidogrel, angiotensin-renin systeminhibitors, and beta-blockers, %	95

CAD: coronary artery disease; PCI: percutaneous coronary intervention; LAD: left anterior descending coronary artery; LCx: left circumflex coronary artery; RCA: right coronary artery; CPK: creatinin phosphokinase.

**Table 2:** Echocardiography and CMR data.

	Baseline	3-monthsfollow-up	
Parameter	Mean±SD	Mean±SD	
**Echocardiographic data**			
LVEDV index (mL/m^2^)	75.3 (60.7;90.4)†		
LVESV index (mL/m^2^)	34.9 (24.9;46.4)†		
WMSI	1.4±0.2		
LVEF (%)	51.2±7.3		
E/E’	10 (8;13)		
Deceleration time ofE (ms)	207±43		
LA volume (mL)	40 (26.6;49.8)		
Sphericity index	1.6±0.2		
MR grade 1/ 2 /3/4 (%)	24/2/0/0		
GLS (%)	−13.9±3.4		
CGS (%)	−14.5±3.6		
RGS (%)	32.7±11.7		
**CMR data**			
LGE mass (g)	17.5 (12.0;35.0)	14.0 (7.5;24.5)*	
TransmuralInfarction % (n)	63 (26)	34 (14)*	
LVEDV index (mL/m^2^)	83.4 (72.9;96.3)	80.6 (69.2;93.9)	
LVESV index (mL/m^2^)	38.6 (34.1;54.5)	34.0 (27.0;51.0)*	
LVEF (%)	50 (45.0;55.5)	57.0 (47.5;62.0)*	
LVM index (gr/m^2^)	62.9±10.9	56.0±11.0*	
MVO mass (g)	2.4±2.5		

LV: left ventricular; LVEDV: LV end-diastolic volume; LVESV: LV end-systolic volume; WMSI: wall motion score index; LVEF: LV ejection fraction; LVM: LV mass; E: E peak velocity on trans-mitral Doppler; E’: E peak velocity on tissue Doppler at the mitral annulus; LA: left atrium; MR: mitral regurgitation; GLS: longitudinal global strain; CGS: circumferential global strain; RGS: radial global strain; LGE: late gadolinium enhancement.

* signifies p<0.05 when comparing baseline and 3-month CMR results (McNemar or Wilcoxon test).

† signifies p<0.05 when comparing baseline global functional parameters as assessed by TTE and CMR (Wilcoxon test).

### Feasibility and Reproducibility of 2D Strain Analysis

No patient was excluded from analysis due to insufficient apical acoustic windows. Longitudinal global strain data was assessed in 40 (97%) patients, and radial and circumferencial strain data in 35 (85%).

**Figure 3:**
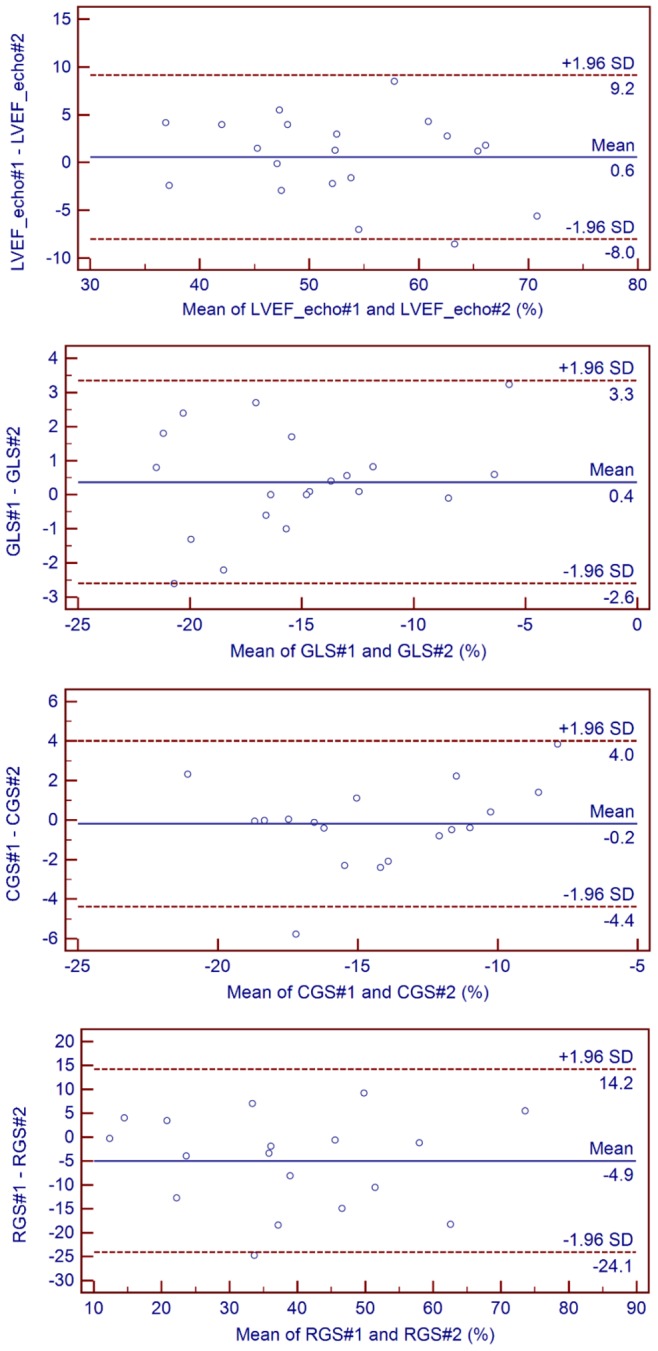
Interobserver Bland and Altman plots. Echo: echocardiography; LVEF: left ventricular ejection fraction; GLS: global longitudinal strain; RGS: radial global strain; CGS: circumferential global strain; #1 for observer 1 and #2 for observer 2; SD: standard deviation.

**Figure 4:**
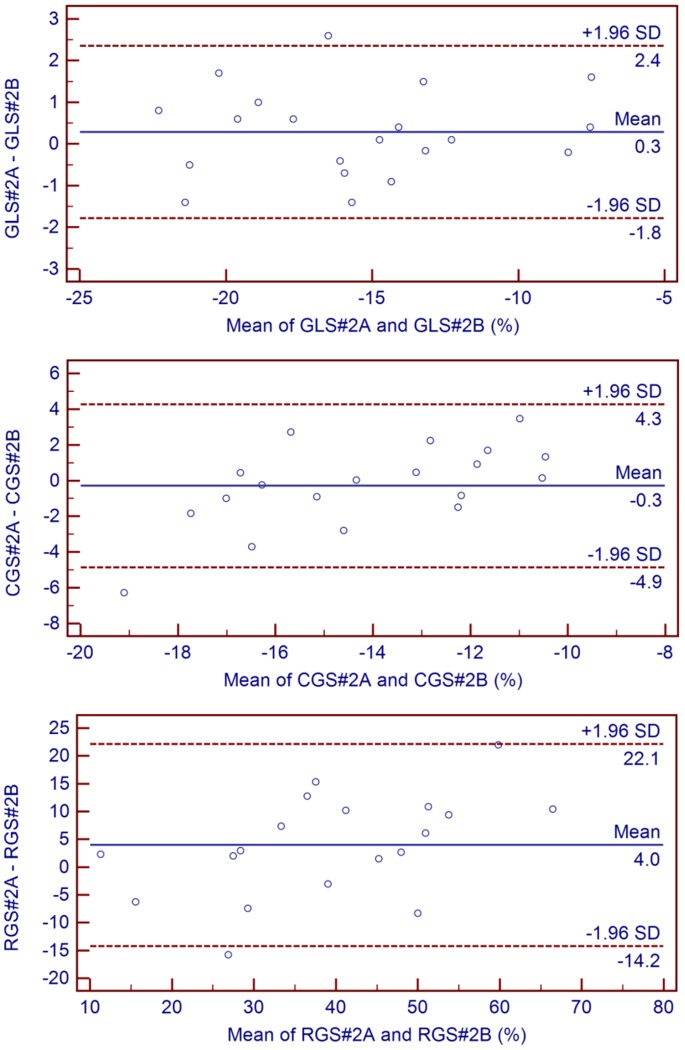
Intra-observer Bland and Altman plots. GLS: global longitudinal strain; RGS: radial global strain; CGS: circumferential global strain; #2A for first measurement and #2B for second measurement; SD: standard deviation.

Bland and Altman tests for longitudinal, radial, and circumferential global strains and LVEF are illustrated in Figures 3 and 4. While longitudinal strain was the most reproducible method (ICC 0.97 [0.93;0.99]), circumferential (ICC 0.92 [0.78;0.97]), and (ICC 0.90 [0.71;0.96]) radial strains were sufficiently reproducible to be integrated in the afterward analysis. Excellent inter-observer agreements for CMR parameters were achieved with the corresponding ICC coefficients as follows: LVEF (0.99 [0.98;1.00]), LVEDV index (0.99 [0.99;1.00]), LVESV index (0.99 [0.98;1.00]), LGE mass (0.99 [0.98;1.00]), and MVO mass (0.99 [0.98;1.00]).

### Correlations between Early Echocardiographic Assessment and Infarct Size as Measured by CMR at 3-month Follow-up

LGE mass evaluated at 3 months significantly correlated with the following echocardiographic data: longitudinal global strain (GLS) (R = 0.601, p<0.001), radial global strain (RGS) (R = −0.405, p = 0.010), circumferential global strain (CGS) (R = 0.526, p = 0.001), LV EF (R = −0.699, p<0.001), WMSI (R = 0.539, p = 0.001), and left atrial volume (R = 0.510, p<0.001).

In multivariate analyses, as illustrated in Table 3, baseline LVEF and GLS were independent predictors of LGE mass at 3 months (respectively, p = 0.004 and p = 0.033).

**Table 3:** Multivariate analysis of echographic data for predicting CMR-derived infarct size (n = 35).

variable	Linear correlationcoefficient	P value	Linear regressioncoefficient ß	Confidence Intervals	P value
GLS	0.601	<0.001	0.877	0.078 −1.679	0.033
RGS	−0.405	0.010			
CGS	0.526	0.001			
LVEF echo	−0.699	<0.001	−0.653	−1.077 - −0.229	0.004
WMSI	0.539	0.001			
LA volume	0.510	0.001	0.195	−0.015–0.405	0.068

GLS: global longitudinal strain; RGS: radial global strain; CGS: circumferential global strain; LVEF echo: LVEF obtained with echocardiography; WMSI: wall motion score; LA: left atrium.

When studying transmural extension of LGE at 3 months, LVEF and CGS were related to LGE above 75% of transmural extension (respectively, p = 0.002 and p = 0.037).

### Echocardiographic Assessment of MVO Measured by CMR at Baseline

MVO was reported in 24 patients (58.5%), and MVO mass significantly correlated with the following echocardiographic data: GLS (R = 0.376, p = 0.010), WMSI (R = 0.387, p = 0.011), and EF (R = −0.389, p = 0.011).

In multivariate analyses, GLS was the only independent predictor of MVO mass (Table 4).

**Table 4:** Multivariate analysis for predicting MVO mass (n = 35).

variable	Linear correlationcoefficient	p value	Linear regressioncoefficient ß	Confidence Interval	P value
GLS	0.376	0.01	0.403	0.071–0.605	0.015
RGS	−0.259	0.06			
WMSI	0.387	0.011			
LVEF echo	−0.389	0.011			

GLS: global longitudinal strain; RGS: radial global strain; WMSI: wall motion score; LVEF echo: LVEF obtained with echocardiography.

### Segmental Analysis and Follow-up Functional Prognosis

Mean GLS was −13.9±3.4%. Mean LS in the remote and infarcted segments was −15.7±3.7% and −10.5±4.6%, respectively. Concerning the infarcted myocardium, mean LS of viable and non viable segments was −14.8±4.6% and −9.1±4.7%, respectively.

Overall, 23 patients presented an akinesia affecting at least three segments at baseline, 10 having recovered at 3-month follow-up.

**Figure 5:**
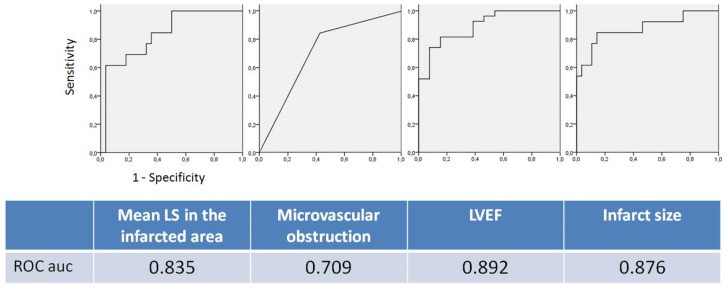
Nonparametric ROC plots of several imaging baseline parameters to the presence of a 3 segment akinesia at follow-up.

ROC curves for predicting akinesia ≥3 segments at follow-up are displayed in Figure 5. A cut-off of infarct LS >−6.0% exhibited a 96% specificity for this prediction (Figure 5 & Table 5). The association of infarct LS >−6.0% with a MVO extending over two segments increased sensitivity and specificity (Table 5).

**Table 5:** Imaging parameters for the prediction of an akinesia affecting at least 3 segments at follow-up.

	Sensitivity (%)	Specificity (%)	PPV (%)	NPV (%)
Infarct LS>−6.0%	61	96	89	84
MVO	57	85	48	89
MVO over 2 segments	54	100	100	82
Infarct LS>−6.0% AND/OR MVO over 2 segments	85	96	92	93

LS: longitudinal strain; MVO: microvascular obstruction.

## Discussion

In the present study, 2D-strain echocardiography conducted within the first days after successful reperfusion in STEMI patients was feasible and provided relevant prognostic information. GLS was a marker of 1) MVO mass assessed a few days after reperfusion and 2) infarct size at 3-month follow-up, while 3) infarct LS >−6.0% measured at the acute phase showed good specificity for diagnosis of a persistent akinesia (≥3 segments) at 3-month follow-up but a poor sensitivity (61%).

Cardiac imaging techniques at the acute myocardial infarction phase mainly aim to provide prognostic factors. Infarct size is well-recognised as a strong outcome predictor following myocardial infarction [21], [22], while the evaluation of transmural myocardial infarct extension is predictive of myocardial viability [23]. CMR performed a few weeks after AMI occurrence allows for quantifying these two prognostic factors using LGE [12], [24]. When LGE-CMR is performed earlier, *i.e.,* a few days after reperfusion, the examination is instrumental in detecting and quantifying MVO [10], [14]. All these factors are predictive of LV remodeling, which is associated with poorer outcome [25]. Echocardiography, as a bedside tool, is able to provide prognostic information at the acute myocardial infarction phase [2], [26], and LVEF and WMSI are the main echocardiographic parameters strongly predictive of all-cause mortality following AMI [27], [28]. In the majority of cases, LVEF is assessed using the Simpson’s biplane method [29]. However, this measurement is a global LV function evaluation using a volumetric approach, and its reproducibility requires training and experience. In contrast, WMSI is rather a regional function parameter, and its evaluation is subjective. Cardiac mechanics are complex, and these two parameters do not allow us to study the spatial organization of myocardial fibers [30]. While longitudinal or long-axis function is mainly accounted for by sub-endocardial layers organized in an oblique clockwise orientation, short-axis function, reflecting the thickening of the myocardium visible in 2D-echocardiography, is mainly due to circumferentially oriented mid-layers and an outer layer arranged in an oblique anticlockwise direction.

New echocardiographic modalities, such as strain imaging, provide new insights into cardiac mechanics, with 2D-strain echocardiography being a highly promising tool [16]. This angle-independent echocardiographic method has been validated against sonomicrometry in animals and tagged-CMR in humans for measuring myocardial deformation [31]. Longitudinal, radial, and circumferential strain can be quantified, reflecting these different deformations. Longitudinal strain has been shown to be the first altered function in AMI due to early necrosis of sub-endocardial layers of myocardial fibers [32], while radial function is still preserved. It has been demonstrated that circumferential strain allows investigators to differentiate between sub-endocardial and trans-mural infarction [9], [33]. Thus, though load-dependent, these parameters provide a more accurate insight into regional myocardial function and its impact on global function.

GLS has recently been studied in AMI, being shown to exhibit a predictive value for infarct size and LVEF recovery [6], [34]–[38]. Several studies have demonstrated the feasibility of measuring these parameters in the AMI setting [5], [39]. Our study confirmed GLS’s value as a marker of infarct size in AMI patients, as evaluated by CMR at 3 months. We studied strain parameters in the three main axes (radial, longitudinal, and circumferential), whereas most other published studies were chiefly focused on longitudinal strain alone [36]. Reproducibility and feasibility of GLS have been well-established in the AMI setting [5], [6], [34], [35], [39], which underlines the parameter’s value as a prognostic tool. However, it appears crucial to also assess radial and circumferential strains on account of the complex organisation and interaction between these deformation parameters [7]. Therefore, in our prospective study comparing CMR and echocardiography, baseline circumferential strain provided information on the transmural extension of infarction. This clinically relevant observation is concordant with results obtained in chronic ischemic cardiomyopathy [9], [15], [33]. In the infarction setting, radial strain is conserved and has thus less usefullness. In the recent VALIANT echocardiographic sub-study, both longitudinal and circumferential strains were predictors of outcomes, whereas only circumferential strain was predictive of remodeling, suggesting that preserved circumferential function might help restrain ventricular enlargement after AMI. [7].

Our CMR study provided additive information on MVO, another post-MI prognostic factor, as GLS was shown to significantly correlate with MVO mass. To the best of our knowledge, MVO’s influence on myocardial strain is still unclear, although MVO was shown to exhibit a significantly negative impact on regional function at the segmental level [40].

## Limitations

This was a prospective, monocentric study, with echocardiography and CMR required to be performed within the first days after reperfusion. Therefore, including a large patient number proved difficult, and the main limitation of our study is its small sample size, essentially due to limited CMR accessibility. As a consequence, we were not able to assess initial strain imaging values so as to predict later LV remodeling, although encouraging results were obtained with surrogate markers, notably myocardial infarct size and MVO.

Our study was exclusively based on 2D strain analysis, itself limited to the quality of view acquisition and to its 2D nature. In-plane and out-of-plane motion may, in fact, pose a problem for strain analysis, which not encountered when using the 3D or MR-tagging approach [41].

It is worth mentioning that although the cut-offs and corresponding sensitivity, specificity, and predictive negative and positive values were specific to our dataset, they were nevertheless of particular interest in terms of defining how GLS relates to akinetic territory. However, when computing the limits of agreements for GLS (see figure 3) and the differences in GLS between viable and not viable segments in infracted myocardium, it should be cautioned that this thresholds may be insufficiently precise for an individual assessment.

## Conclusion

2D-strain echocardiography carried out as a bedside test within a few days after reperfusion in STEMI patients provides indices similar to those obtained using CMR. Further studies involving larger patient series and registries are needed to confirm the parameters’ prognostic value in terms of LV remodeling and clinical patient outcome.
